# Application of alignment-free bioinformatics methods to identify an oomycete protein with structural and functional similarity to the bacterial AvrE effector protein

**DOI:** 10.1371/journal.pone.0195559

**Published:** 2018-04-11

**Authors:** Devdutta Deb, David Mackey, Stephen O. Opiyo, John M. McDowell

**Affiliations:** 1 Department of Plant Pathology, Physiology and Weed Science, Virginia Tech, Blacksburg, Virginia, United States of America; 2 Departments of Horticulture and Crop Science and Molecular Genetics, Ohio State University, Columbus, Ohio, United States of America; 3 Molecular and Cellular Imaging Center-Columbus, Ohio Agricultural Research and Development Center, Ohio State University, Columbus, Ohio, United States of America; Agriculture and Agri-Food Canada, CANADA

## Abstract

Diverse plant pathogens export effector proteins to reprogram host cells. One of the most challenging goals in the molecular plant-microbe field is to functionally characterize the complex repertoires of effectors secreted by these pathogens. For bacterial pathogens, the predominant class of effectors is delivered to host cells by Type III secretion. For oomycetes, the predominant class of effectors is defined by a signal peptide that mediates secretion from the oomycete and a conserved RxLR motif. Downy mildew pathogens and *Phytophthora* species maintain hundreds of candidate RxLR effector genes in their genomes. Although no primary sequence similarity is evident between bacterial Type III effectors (T3Es) and oomycete RXLR effectors, some bacterial and oomycete effectors have convergently evolved to target the same host proteins. Such effectors might have evolved domains that are functionally similar but sequence-unrelated. We reasoned that alignment-free bioinformatics approaches could be useful to identify structural similarities between bacterial and oomycete effectors. To test this approach, we used partial least squares regression, alignment-free bioinformatics methods to identify effector proteins from the genome of the oomycete *Hyaloperonospora arabidopsidis* that are similar to the well-studied AvrE1 effector from *Pseudomonas syringae*. This approach identified five RxLR proteins with putative structural similarity to AvrE1. We focused on one, HaRxL23, because it is an experimentally validated effector and it is conserved between distantly related oomycetes. Several experiments indicate that HaRxL23 is functionally similar to AvrE1, including the ability to partially rescue an AvrE1 loss-of-function mutant. This study provides an example of how an alignment-free bioinformatics approach can identify functionally similar effector proteins in the absence of primary sequence similarity. This approach could be useful to identify effectors that have convergently evolved regardless of whether the shared host target is known.

## Introduction

Successful plant pathogens must overcome a multilayered system of inducible immune responses that plants have evolved to defend against pathogen attack [1, 2]. The first layer of immunity, termed pattern-triggered immunity (PTI, [3]), is activated when conserved microbial molecules (pathogen-associated molecular patterns or PAMPs) are recognized by pattern recognition receptors (PRRs) in the host [4]. This recognition event triggers a complex immune response that includes the production of reactive oxygen species (ROS), deposition of callose and phenolic compounds in the cell wall, and extensive reprogramming of the transcriptome. Plant pathogenic bacteria, fungi, oomycetes, nematodes, and insects secrete effector proteins that suppress this immunity by directly targeting the PRRs or manipulating regulatory hubs in the PTI signaling network [5].

The second layer of plant immunity is referred to as effector-triggered immunity (ETI, [6]). ETI is based on direct or indirect recognition of pathogen effectors by corresponding plant immune surveillance proteins, known as resistance or “R” proteins [7, 8]. This recognition triggers a robust and effective suite of defense responses that typically includes the hypersensitive response (HR), featuring programmed cell death at the site of infection [9]. Pathogens, in turn, have shuffled their effector repertoire or evolved novel effectors to counteract ETI by either avoiding R protein recognition or suppressing downstream signaling events [10, 11].

In addition to suppressing host defense, bacterial and perhaps other classes of pathogens deploy effectors to control the extracellular plant environment. Availability of suitable nutrients and water are essential to pathogen growth and induction of disease. Numerous T3Es of the Transcriptional Activator-Like (TAL) family control nutrient availability through direct or indirect transcriptional induction of SWEET-family sugar transporters [12–15]. Other T3Es promote the conversion of the plant extracellular space into an aqueous environment [16]. “Water-soaking” is a classic symptom in plants infected with phytopathogenic bacteria that might benefit the bacteria in various ways, including to offset plant defenses associated with limited water availability during the HR [17].

The virulence-promoting activities of effectors are best understood in bacteria, owing to the focus on genetically tractable model strains such as *Pseudomonas syringae* strain DC3000 [18]. The predominant category of effectors in phytopathogenic bacteria are delivered to the interior of host cells via Type III secretion [19]. Significant progress has been made in elucidating the targets and establishing the functions and targets of bacterial T3Es [5]. In this study, we focused on the highly conserved and widespread AvrE-family of T3Es. AvrE-like proteins are encoded by *Pseudomonas*, *Pectobacterium*, *Erwinia*, *Pantoea* and *Dickeya*. AvrE1 from *P*. *syringae* belongs to a so-called redundant effector group (REG) comprised of AvrE1, HopM1, and HopR1. AvrE1 resides within the conserved effector locus (CEL) region that also harbors HopM1, HrpW and HopAA1. Conservation of the CEL, and especially an AvrE-family T3E, in diverse genera of bacteria [20] indicates the importance of both the locus and the AvrE-family T3Es.

T3Es of the AvrE-family display each of the general activities discussed above, including elicitation of ETI in nonhost plants and suppression of PTI and elicitation of water-soaking in host plants [21–27]. The AvrE-family effectors are large proteins with low sequence identity. For example, WtsE, the AvrE-family effector protein from the maize pathogen *Pantoea stewartii* pv. *stewartii*, shares 27.1% amino acid identity with AvrE1 from *P*. *syringae* [21]. AvrE-family effectors share WxxxE motif and a C-terminal endoplasmic reticulum membrane retention/retrieval signal (ER-MRS), which were required for activities of AvrE1 and WtsE in both host and nonhost plants [21]. The ability of both AvrE1 and WtsE to carry out their virulence activities in host plants depends on their interaction at the plasma membrane with specific regulatory subunits of the heterotrimeric protein phosphatase, PP2A [28, 29]. Host proteins whose phosphorylation status is affected by the interaction of the AvrE-family T3Es with PP2A remain to be identified. AvrE1 has recently been linked to water-soaking during infection of *Arabidopsis* [16], but it is unknown whether this function requires interaction with PP2A subunits.

Oomycetes are filamentous eukaryotic microorganisms, many species of which cause important diseases of crops. Oomycetes secrete effector proteins outside and inside host cells to promote infection and colonization [30–32]. Recently published genome sequences of species from some of the important oomycete genera including *Phytophthora* [33–35], *Pythium* [36], *Albugo* [37, 38] and *Hyaloperonospora* [39] revealed large repertoires of candidate effectors in these pathogens. A major class of effectors in *Phytophthora* species and downy mildew pathogens is named “RxLR”. These proteins are defined by an N-terminal signal peptide (SP), followed by the conserved motif, RXLR (where R is Arginine, X is any amino acid and L is leucine). The RXLR motif has been proposed to specify translocation to the interior of host cells [40, 41], but this is controversial [42, 43]. Another family of oomycete effector proteins are named “CRN” for the crinkling and necrosis phenotype that some family members induce when transiently expressed in leaves [44]. These proteins are defined by an N-terminal SP followed by a LXLFLAK motif, and appear to be translocated to the interior of host cells. Virulence-promoting functions have been ascribed to some members of this family [45].

A major area of current interest is to more thoroughly understand how RxLR proteins promote oomycete virulence [46]. This challenge is compounded by two factors: First, RxLR gene families range in size from 130 to over 550 amino acids [32]. Second, almost all of the RxLR effector genes encode novel proteins with no recognizable functional motifs [46]. Thus, new computational approaches for predicting RxLR protein function are needed [47]. A major premise of this study is that it would be useful to leverage information from the study of bacterial type III effectors to frame hypotheses about the function of RxLR effector proteins. Because effectors from bacteria and oomycetes share common, *in planta* targets [48, 49], convergent evolution may have given rise to effector domains that share no discernable primary sequence similarity, but share similar chemical/structural features that facilitate shared virulence-promoting functions. Such domains could be revealed through the use of recently developed bioinformatic tools that predict structural similarity in unrelated proteins. Partial least squares (PLS) is a regression method that is trained to learn from positives samples (example, AvrE), and negative samples (example, Non-AvrE) for future predictions. We reasoned that this approach could be used to identify oomycete effectors with putative structural similarity to well-studied bacterial effectors such as AvrE. Such effectors would represent potentially promising leads for downstream functional analysis.

As a first step towards testing the validity of this approach, we applied a PLS alignment-free bioinformatics method to identify oomycete RxLR effectors with structural similarity to bacterial AvrE-family type III effectors. We focused on the oomycete *Hyaloperonospora arabidopsidis* (*Hpa*), a naturally occurring downy mildew pathogen of *Arabidopsis* [50]. The genome of a reference *Hpa* isolate has been sequenced and 134 high-confidence RXLR genes have been annotated [39]. Several Hpa RXLR effectors have been functionally characterized [39, 46]. The PLS alignment-free methods identified five candidate RxLR proteins and four CRN proteins from the *H*. *arabidopsidis* genome that are similar to AvrE-family proteins. We predicted the structure of one of the identified proteins (HaRxL23 [51]) and AvrE1 from *P*. *syringae* using the iterative threading assembly refinement (I-TASSER [52–54]), and compared their structural similarity using the DaliLite [55] method. Based on this similarity, we hypothesized that AvrE1 and HaRxL23 may have convergently evolved to perturb the same host target(s). As an initial test of this hypothesis, we examined the activity of HaRxL23 and AvrE1 in a variety of biological assays and observed the two proteins to be functionally similar.

## Materials and methods

### Bioinformatics analysis

#### Data sets

Twelve AvrE proteins (positives) from the study by Ham and associates [21] and non-AvrE proteins (negatives) were downloaded from National Center for Biotechnology Information (NCBI) websites (http://www.ncbi.nlm.nih.gov/), and were used for training the PLS methods. The *H*. *arabidopsidis* RxLR candidate gene sequences used in this study were as described in Baxter et al. [39]. Accession numbers for all sequences are listed in S1–S3 Tables.

#### Amino acid composition

From each protein sequence, frequencies of 20 amino acids were calculated. In this study, amino acid composition was used as descriptors for a PLS classifier (PLS-AA).

#### Dipeptide composition

Dipeptide composition represents all 400 frequencies of consecutive amino acid pairs in a protein sequence and corresponds to a 400 (20 X 20) feature vector. It can encapsulate information on composition of amino acids as well as their local order. We used dipeptide composition as descriptors for a PLS classifier (PLS-DIP).

#### Physicochemical properties of amino acids

In Opiyo and Moriyama [56], we developed five descriptors (the first five principal component scores [PC1- PC5]) using the principal component analysis (PCA) of 12 physicochemical properties of amino acids (mass, volume, surface area, hydrophilicity, hydrophobicity, isoelectric point, transfer of energy solvent to water, refractivity, non-polar surface area, and frequencies of alpha-helix, beta-sheet, and reverse turn). The five descriptors (PC1-PC5) were used in this study.

#### Auto/Cross covariance transformation

Auto/cross covariance (ACC) transformation method discussed in [56] was used to transform each amino acid sequence using the five descriptors (PC1-PC5). ACC with the maximum lag of 30 residues yielded 775 descriptors for each sequence. The calculation of ACC was performed using the R implementation (version 2.12.0; http://www.R-project.org; 2010).

#### Partial least squares

As described in detail previously [57] partial least squares (PLS) is a regression used for predictions. PLS is a projection method similar to principal component analysis (PCA) where the independent variables, represented as the matrix **X**, are projected onto a low dimensional space. PLS uses both independent variables **X** (sequence descriptors such as amino acid composition) and dependent variables **Y** (positive or negative label) [58]. PLS using descriptors transformed by ACC (PLS-ACC) was used in [56]. PLS discriminant analysis is performed to separate groups of observations. It consists of a classical PLS where the response variable is a categorical one (replaced by the set of dummy variables describing the categories, e.g., 0 and 1) expressing the class membership of the samples. In this study, each of a training sample, a response variable was assigned 1 for the positive sample (AvrE) and 0 for a negative sample (non-AvrE). The group membership, AvrE or non-AvrE of a new sequence was predicted based on descriptors and y-value. Predicted y-value closer to 1 was considered to be AvrE candidate and closer to 0 considered to be non-AvrE candidate. PLS analysis was performed using an R implementation, the PLS package developed by Wehrens and Mevik (version 1.2.1 [59]).

#### Performance analysis

Cross-validation analysis (leave-one-out) was performed for all the 24 sequences used for training the methods. One sequence in the training dataset was left out and the learning algorithm was trained on the rest of the sequences. The trained model was used to predict the class (AvrE or non-AvrE) of the proteins left out of the training. For the 24 sequences, the process was repeated 24 times leaving each of the 24 sequences out and creating a model from the remaining 23 sequences.

Predictions were grouped as follows:

- True Positives (TP): the number of actual AvrE proteins (AvrEs) that were predicted as AvrEs.
- False Positive (FP): the number of actual non-AvrEs that were predicted as AvrEs.
- True Negative (TN): the number of actual non-AvrEs that were predicted as non-AvrEs.
- False Negative (FN): the number of actual AvrEs that were predicted as non-AvrEs

#### Minimum error point

The minimum error point [60] was used to determine threshold values of PLS methods. The sequences are ranked based on the values. The threshold value where the minimum number of errors (FN + FP) occurs is the minimum error point (MEP) and the number of false positives and false negatives are assessed at this point. The minimum error point reports the best-case accuracy of a method. The minimum error points for PLS-AA, PLS-DIP, and PLS-ACC were 0.94, 0.96, and 0.94, respectively. The upper cut-off point for all methods was set at 1.00 to further reduce the number of false positives. To be selected as a candidate, a protein has to be identified by all the three methods (PLS-AA, PLS-DIP, and PLS-ACC) as positive.

#### Goodness of prediction of PLS methods

The goodness of prediction, q^2^ Eq 1, describes how well the method can predict a data.

$$q^{2}=1-\frac{\sum_{i=1}^{N}{\left(Y_{a}-Y_{p}\right)}^{2}}{{\sum_{i=1}^{N}\left(Y_{a}-Y_{m}\right)}^{2}}$$ (1)

where *Y*_a_ is a sample from a training data, *Y*_p_ is the omitted sample, *Y*_m_ is the mean of the samples of the training data, and *N* is the total number of samples in the training data. The value of q^2^ > 0.50 is considered good. In this study, the leave-one-out cross-validation procedure was used for the q^2^ calculation. Detailed results of PLS analyses, and the *H*. *arabidopsidis* proteins identified by each method, are given in Tables 1 and 2 and S4 Table for PLS-AA, PLS-DIP, and PLS-ACC, respectively.

**Table 1:** The number of PLS components and the predictive abilities of PLS-AA, PLS-DIP, and PLS-ACC, respectively from the leave-one-out cross validation procedures.

Methods	Number of PLS components	q^2^
PLS-AA	4	0.72
PLS-DIP	3	0.67
PLS-ACC	4	0.78

**Table 2:** Nine protein candidates identified from *Hyaloperonospora arabidopsidis* genome by the three methods.

Accession number	PLS-AA	PLS-DIP	PLS-ACC
HaRxL23	0.98	0.99	0.95
HaRxL33	0.99	1.00	1.00
HaRxL71	1.00	0.98	0.94
HaRxL94	0.94	0.96	0.94
HaRxL120	0.94	0.97	1.01
HaCRN9	0.95	0.99	1.00
HaCRN10	0.97	1.00	1.00
HaCRN12	0.98	0.97	0.95
HaCRN14	0.94	0.96	0.98

#### Structural prediction of AvrE1 and the nine predicted proteins by I-TASSER, and structural comparison by DaliLite

To predict the structures of AvrE1 and nine predicted protein candidates from *H*. *arabidopsidis*, we selected the I-TASSER server because it predicts secondary and tertiary structures, and ligand-binding sites [52–54, 61]. After the predictions, the structural similarity of AvrE1 and the nine protein candidates were compared using DaliLite [62] (S5 Table). The structures of the DspA/E propeller-domain amino acids and the AvrE1 1513–1578 region were predicted using I-TASSER (structural templates are listed in S6 Table). The predicted structures of DspA/E and AvrE1 region were aligned using TM-align [63].

### Experimental comparisons of HaRxL23 and AvrE1

#### Construction of expression plasmids

*HaRxL23* was amplified from genomic DNA extracted from *Arabidopsis* Oy-1 plants, infected with *Hpa* isolate *Emoy2*, using proofreading polymerase (Pfu, Invitrogen). Forward and reverse primers were designed to amplify from the signal peptide cleavage site (HaRxL23 NOSP, S7 Table) with or without the stop codon (HaRxL23 S and HaRxL23 NS, respectively, S7 Table) depending on the type of fusion. For cloning into Gateway destination vectors, the sequence CACC was added at the 5’ end of the forward primer and PCR was performed using the genomic DNA as template. PCR products were gel purified (Qiagen) and recombined into pENTR-D-TOPO Gateway entry vector following the manufacture’s protocol (Invitrogen). This step was followed by transformation into *Escherichia coli* DH5α competent cells. Kanamycin resistant colonies were selected on agarose plates followed by colony PCR with plasmid-specific M13 forward and reverse primers. Colonies having the correct size were selected for plasmid purification and confirmed by sequencing. The pENTR clone generated was then used to create Gateway expression plasmids using LR recombination (Invitrogen).

For *Pseudomonas*-mediated transient studies, the HaRxL23 gene was shuttled from pENTR into pEDV6 by LR recombination. pEDV6 contains the *AvrRPS4* promoter and leader sequence that directs the fusion protein through the Type III secretion system [64]. The EDV constructs with our effectors were transformed into *Pseudomonas phaseolicola* strains by standard tri-parental mating using *E*. *coli* pRK600 as a helper strain. The *Pph* 3121, *Pph* AvrE1, *ΔavrE1* mutant (CUCPB5374) and *ΔavrE1*(*avrE1*) (pCPP5246) strains have been previously described [65, 66]. All gene fusions were confirmed by sequencing.

#### Plant materials and growth conditions

*Arabidopsis* and tomato (*Lycopersicon esculentum* cv. Moneymaker) plants were grown in Sunshine Pro-mix soil mixture number one. For experiments involving inoculation with *Pseudomonas spp*., *Arabidopsis* was grown in controlled growth chambers under short day cycles (8h/16h light/dark and 150–200 μE/m^2^s) at 22°C and 60% relative humidity. For all other experiments, *Arabidopsis* and tomato were grown under long day cycles (16h/8h light/dark at 90–100 μE/m^2^s) at 22°C and 60% relative humidity.

#### Assays for plant cell death, bacterial virulence and callose suppression in *Arabidopsis*

For assays involving *Pseudomonas spp*., *Arabidopsis* Col-0 plants were syringe-infiltrated with 1x10^5^ colony-forming units (cfu)/ml (virulence assays) or 1x10^8^ cfu/ml (cell death and callose suppression assays) bacterial solution in 10mM MgSO_4_. For cell death assays, a total of 6 plants, 3 leaves each, were infiltrated and visual scoring was performed 16–20 hours later. For bacterial growth assays, leaf discs were scored at zero and three days post infiltration (dpi), surface sterilized with 70% ethanol, and homogenized using a mini-bead beater (Biospec products). Serial dilutions were performed to count colony-forming units. For each sample, three leaf discs were pooled three times per data point. For callose suppression assays, whole leaves were harvested 16 hours post infiltration (hpi), treated with alcoholic lactophenol and stained with 0.01% (w/v) Aniline blue stain in K_2_HPO_4_ buffer as described previously [64]. Stained leaves were mounted on glass slides using 50% glycerol and imaged with a Zeiss Axio Imager.M1 using the filter settings for DAPI. Quantification of callose spots was performed using the Autospots software [67]. Statistical analyses for growth curves were performed on the means of log-transformed data using Student’s t-test (*p < 0.01, **p <0.001).

#### Bacterial lesion assay in tomato Moneymaker plants

For the bacterial lesion assay, 4–5 week old *Solanum lycopersicum* cv. Moneymaker (tomato) plants were dip-inoculated for 30 seconds with 1x10^8^ cfu/ml bacterial solution in 10mM MgSO_4_ containing 0.02% Silwet. A total of 3–4 plants were used for each treatment. Disease symptoms on leaves in the form of small, brown, necrotic lesions were monitored for a total of six days. At five days after inoculation, the number of well-developed lesions (≥0.25 mm^2^) per leaf was quantified.

## Results

### Mining *Hyaloperonospora arabidopsidis* candidate RXLR proteins using three PLS methods

In this study, we trained three PLS methods (PLS-AA, PLS-DIP, and PLS-ACC) using AvrE family proteins to identify similar RxLR effector proteins from *H*. *arabidopsidis* RXLR proteins. The scores ranged between 0 and 1, with the cut-off point of 0.5 for AvrE candidates, and less than 0.5 for Non-AvrE candidates. The higher the score, the higher the confidence that the protein is an AvrE candidate. A total of 20 HaRxL proteins, five HaCRN proteins, and four non-effector proteins were identified by at least one of the three PLS methods (S4 Table). Of these, five *Hpa* RxLR protein candidates and four CRN proteins were identified by all three PLS methods with scores > 0.9 (Table 2). Of these nine candidates, only HaRxLR23 has been validated as a *bona fide* effector gene [51].

### Structural predictions of AvrE1 and the nine protein candidates by I-TASSER and comparison with DaliLite

**Fig 1:**
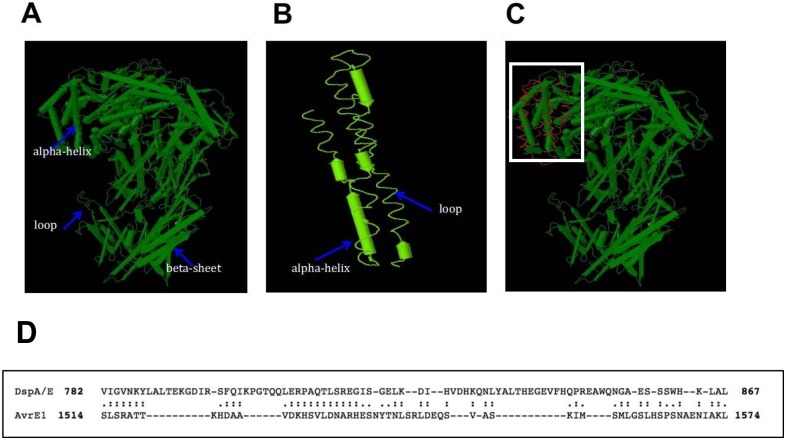
Structural predictions of HaRxL23 and AvrE1. (A) Predicted structure of AvrE1 by I-TASSER (B) Predicted structure of HaRxL23 by I-TASSER (C) Superimposed structure of HaRxL23 on AvrE1 by DaliLite. Structure of HaRxL23 is shown in red. (D) Structural alignment of DspA/E propeller domain and AvrE1 region using TM-aligned. ":" denotes aligned residue pairs of RMSD < 5.0 Angstroms, "." denotes other aligned residues.

We obtained the DaliLite Z-scores from pairwise comparison of the predicted structures of AvrE1 and the nine protein candidates. Five of the nine (HaRxL23, HaRxL33, HaRxL71, HaRxL94, and HaCRN10) had structural similarity with AvrE1. The predicted structures of AvrE1 and HaRxL23 are presented in Fig 1. AvrE1 is a very large protein of 1795 amino acids and HaRxL23 is a small protein with 142 amino acids. Despite this disparity in size and the fact that the proteins share no significant sequence identity, it can be seen that HaRxL23 overlaps structurally with amino acids 1513 to 1578 of AvrE1 (Fig 1). The HaRxL23 ortholog from *Phytophthora sojae*, PsAvh73 [51], overlaps structurally with the same domain of AvrE1 (S1 Fig). This domain of AvrE1 does not have the WxxxE and ER-MRS motifs, but corresponds to the double beta propeller domain of another AvrE-family T3E, DspA/E from *Erwinia amylovora*, for which mutational analysis has indicated functional importance [68].

### HaRxL23 and AvrE1 induce cell death in young *Arabidopsis* plants when delivered by *Pseudomonas phaseolicola* (*Pph*) 3121

Because HaRxL23 has been confirmed as an effector [51] and also had the highest Z-score of 6.7 (S5 Table), we focused on functional comparisons of this protein with AvrE1. Previous studies with AvrE1 of PtoDC3000 and its ortholog in *Pantoea stewartii*, WstE, revealed that both proteins are capable of inducing a cell death response in *Nicotiana tabacum* and tomato plants when secreted by bacteria that are inoculated at a relatively high density [22, 24, 66]. As a first test of functional similarity between HaRxL23 and *Pst* AvrE1, we tested whether HaRxL23 was capable of inducing cell death in *Arabidopsis* when delivered from the bean pathogen, *P*. *syringae pv*. *phaseolicola NPS3121 (Pph)* at a high inoculum dose. We used *Pph* for our experiments because it is non-pathogenic on *Arabidopsis*, has a functional TTSS, and does not elicit cell death or any other symptoms. Thus, the endogenous AvrE homolog in *Pph* NPS3121 does not induce cell death in *Arabidopsis*. We engineered *Pph* to use the “effector detector vector (EDV)” to deliver HaRxL23 via the type III secretion system (TTSS) to the interior of plant cells [64]. The HaRxL23 coding region, beginning downstream of the cleavage site of the signal peptide, was cloned as a translational fusion to the promoter and N-terminal region of the *P*. *syringae* effector AvrRps4. Transgenic *Pph* strains containing this plasmid could translate the fusion protein and secrete it into plant cells via the Type III translocon, enabling the effects of HaRxL23 inside plant cells to be assayed experimentally. This strain was used in comparison with a previously described *Pph3121* strain that delivers *PstDC3000* AvrE1 from a plasmid vector [21].

**Fig 2:**
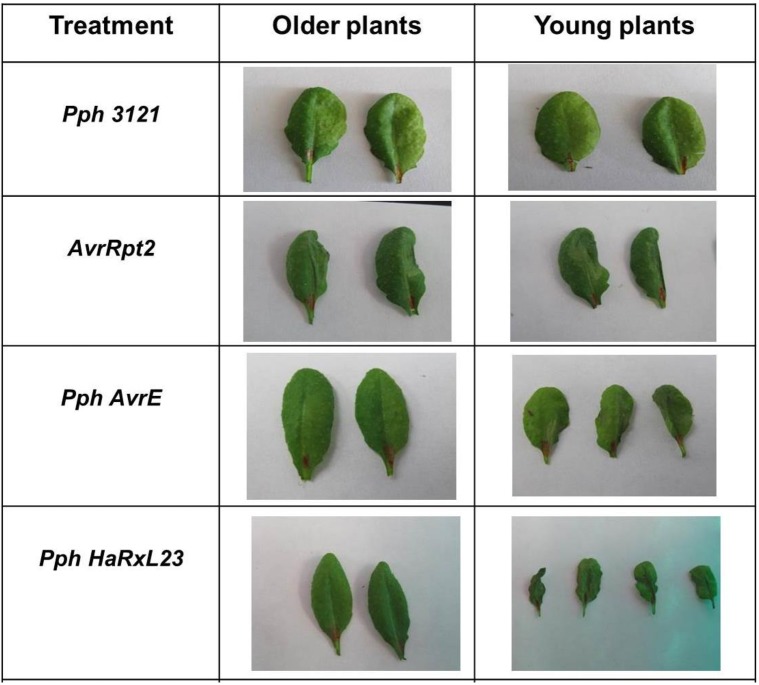
Both HaRxL23 and AvrE1 induce cell death in young *Arabidopsis* plants when delivered by *Pseudomonas phaseolicola*. Images from the cell death test in leaves of *Arabidopsis* wild type plants (Col-0). Five-week old plants and three-week old plants were infiltrated with suspensions of *P*. *phaseolicola* expressing effectors (1 X 10^8^ cfu/ml). Cell death was visually monitored over a period of 20 hours after inoculation. This experiment was repeated three times with similar results.

In accordance with previous results, a typical HR cell death symptom of leaf collapse was observed when the *P*. *syringae* effector AvrRpt2 was delivered into the *Arabidopsis* ecotype Col-0 as a positive control (Fig 2) [64]. Delivery of either HaRxL23 or AvrE1 triggered leaf collapse symptoms comparable to AvrRpt2 in three-week old *Arabidopsis* plants (Fig 2). This response was not seen in five-week old plants (Fig 2). This experiment demonstrates that, when delivered by *Pph*, both AvrE1 and HaRxL23 can elicit cell death specifically in young *Arabidopsis* plants.

### HaRxL23 and AvrE1 suppress callose deposition elicited in *Arabidopsis* by *Pph*

**Fig 3:**
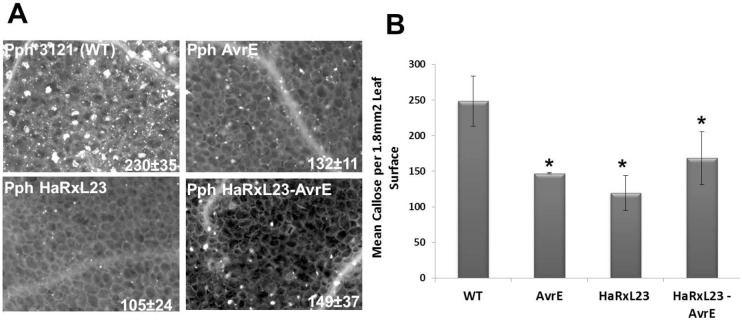
AvrE and HaRxL23 suppress callose deposition in *Arabidopsis* when delivered by *P*. *phaseolicola* via the effector detector vector system. (A) Four-week old *Arabidopsis* Col-0 plants were infiltrated with 5 X 10^7^ cfu/ml *Pph* strains expressing either AvrE1or HaRxL23 individually, or a double transformant expressing HaRxL23 and AvrE1 together. Callose deposits were visualized by staining with aniline blue and (B) quantified using the Autospots software program. Four pictures per leaf from six leaves were analyzed per treatment. P-value * < 0.01; t-test comparisons representing significant differences with Col-0. Error bars represent Standard Error of six independent leaf samples tested at the same time. This experiment was repeated three times with similar results.

Another advantage of using *Pph* is that it also elicits robust defenses in *Arabidopsis*, including deposition of callose and accumulation of the defense marker protein Pathogenesis Related-1 (PR-1). Thus, the system can be used to study the ability of a heterologous effector to suppress the *Pph*-induced defenses. Suppression of cell wall-based defenses (e.g., callose deposition) is considered an important function of both bacterial and oomycete effectors. Callose is a polymer of β-1, 3 glucans that is deposited between the cell wall and cell membrane near the invading pathogen [69]. Such foci are easily visualized by fluorescence microscopy and can be quantified as a readout of host defense [64]. We compared the ability of HaRxL23 and AvrE1 to suppress callose deposition when delivered from *Pph*, by inoculating four-week old plants at a titer that does not induce the cell death response described in Fig 2. As expected, *Arabidopsis* Col-0 plants exhibit extensive callose deposition when syringe-infiltrated with wild type *Pph* 3121 (Fig 3A). Contrastingly, a reduction of close to 50% in callose deposits is observed in plants that were syringe-infiltrated with strains of *Pph* expressing either HaRxL23 or AvrE1 (Fig 3A and 3B). It is interesting to note that this suppression is not enhanced when a double transformant, *Pph* HaRxL23 + AvrE1, is used (Fig 3A and 3B). This lack of additivity indicates that both effectors likely interfere with the same regulatory pathway in the host.

### Neither HaRxL23 nor AvrE1 enhance *Pph 3121* virulence

**Fig 4:**
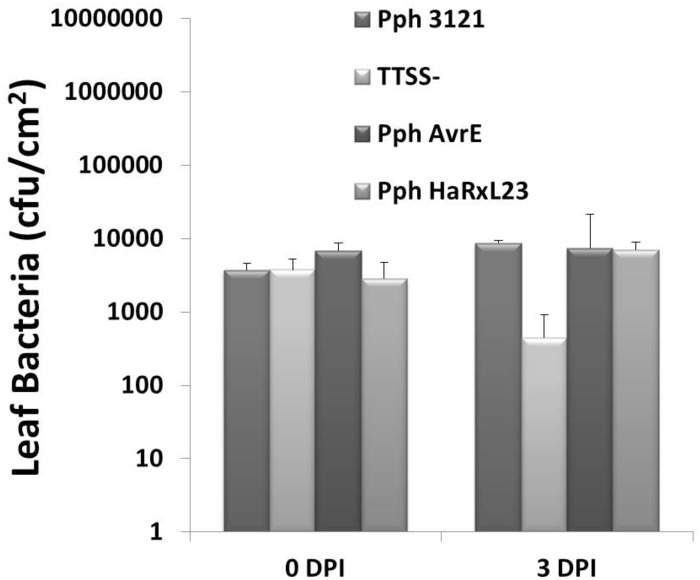
Pph 3121 virulence is not enhanced by transgenic HaRxL23 or AvrE. Plants were infiltrated with a bacterial suspension of 1 X 10^5^ cfu/ml. Bacterial populations were determined at day 0 and day 3 after inoculation. Error bars indicate Standard Error of six independent leaf samples tested at the same time. The experiment was repeated three times with similar results.

The assay in Fig 2 for macroscopic plant cell death was carried out with a high dose of bacterial inoculum (5 X 10^7^ cfu/ml). To test whether HaRxL23 enhances or suppresses bacterial growth *in planta*, we infiltrated *Arabidopsis* leaves with a low dose of bacteria (1 X 10^5^ cfu/ml) and then measured bacterial growth at three days after inoculation. We compared the growth, in Col-0, of *Pph* expressing HaRxL23 and AvrE1 to *Pph* with an empty vector control (Pph3121) and a non-pathogenic mutant deficient in Type III secretion (TTSS-). At three days post-infiltration, there was no enhancement of bacterial growth for either *Pph HaRxL23* or *Pph AvrE1* compared to *Pph 3121 EV* (Fig 4) in young *Arabidopsis* Col-0 plants. Thus, despite the ability of HaRxL23 and AvrE1 to suppress callose elicited by *Pph 3121*, there is no net enhancement of *Pph* virulence in *Arabidopsis* by either effector.

### HaRxL23 can rescue the reduced virulence phenotype of the *PtoDC3000ΔavrE1* strain in tomato Moneymaker

**Fig 5:**
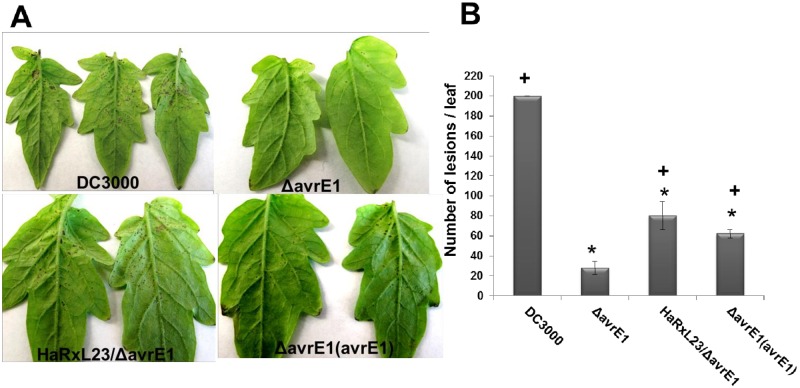
HaRxL23 rescues the reduced lesion phenotype of the ΔavrE1 strain in tomato Moneymaker plants. (A) Disease symptoms (lesion production) on tomato cv. *Moneymaker* plants 8 days after dip inoculation of the indicated strains of *Pto* DC3000 at 1x10^8^ cfu/ml bacterial culture. (B) Number of lesions (≥0.25mm^2^) per whole leaf appearing on plants 8 days after dipping inoculation with the respective bacterial strains. Values indicate mean and error bars indicate standard error on at least five whole leaves assayed for each treatment. P-value * < 0.01; t-test comparisons representing significant differences with DC3000; **+** < 0.01; t-test comparisons representing significant differences with ΔavrE1. This experiment was repeated three times with similar results.

The most stringent genetic test for functional equivalence between *HaRxL23* and *AvrE1* is to assay whether *HaRxL23* can rescue an *AvrE* loss-of-function mutant. However, this experiment is complicated by functional redundancy between *AvrE* and other *Pst* effectors, such that a *ΔavrE1* mutant does not display a phenotype under most previously tested conditions. The only phenotype of an *AvrE1* mutant was reported by Badel et al., who observed that *P*. *syringae* pv. *tomato* DC3000 *ΔavrE1* deletion mutant was impaired in the formation of bacterial speck lesions in tomato (*Solanum lycopersicum* cv. Moneymaker) plants [66]. Thus, we hypothesized that if HaRxL23 is functionally similar to AvrE1, then HaRxL23 would be able to rescue the reduced bacterial speck lesion phenotype of the *avrE1* mutant in tomato. Four-week old tomato Moneymaker plants were dip-inoculated with bacterial suspensions containing wild-type DC3000, the *ΔavrE1* mutant, the *ΔavrE1* mutant carrying *HaRxL23*, and the complemented *ΔavrE1*(*avrE1*) strain (Fig 5). Bacterial speck lesions ≥0.25 mm^2^ were quantified from infected leaves. Like *AvrE1*, *HaRxL23* was able to significantly increase the number of lesions produced by the *ΔavrE1* mutant strain (Fig 5). Interestingly, the virulence of the strain expressing *HaRxL23* was slightly stronger than that observed in the complemented strain of *ΔavrE1* [66], which is known to have a partial rescue phenotype, relative to the wild-type strain, presumably due to inefficient expression of the plasmid-encoded gene.

## Discussion

Over the last three decades, it has become clear that secreted effector proteins are critically important virulence or avirulence factors for essentially every category of plant pathogen and pest. Studies of bacterial effectors have provided seminal insights into the molecular mechanisms that underpin pathogen virulence and host plant resistance [2]. Moreover, bacterial effectors have been used as molecular probes to decipher unknown aspects of plant biology [70, 71]. This is well-exemplified by studies of AvrE-family T3Es, which have uncovered previously unknown plant immune regulators.

Similar studies of effectors from eukaryotic pathogens are progressing quickly, thanks in part to the falling cost of sequencing, enabling draft genomes and transcriptomes to be assembled for many fungi and oomycetes. These resources have been combined with computational tools to enable prediction of candidate effector genes [47]. This is well-exemplified by the use of Hidden Markov Models to identify RxLR genes in oomycete genomes [72]. However, such computational approaches typically yield hundreds or even thousands of candidate genes, particularly for fungal genomes in which no clear consensus motifs, other than a signal peptide, have emerged. Moreover, the primary sequences of effector proteins typically provide no clues to their biochemical functions or molecular targets in plant cells. Thus, a need exists for additional computation tools to enable prediction of molecular functions and prioritization of effector genes for time-consuming molecular studies [47].

With this bottleneck in mind, we connected two recent but disparate advances: The first is emergence of new computational methods for predicting structural similarity between proteins, in the absence of strong similarity in primary sequence. The second is the emerging understanding, from model plant pathosystems, that effectors from divergent pathogen lineages (e.g., bacteria and oomycetes) convergently evolve to target the same points of vulnerability in plant regulatory networks. Most often these targets comprise highly connected regulatory hubs, perturbation of which would have major effects on plant cell signaling [73]. We reasoned that this convergence provides an opportunity to exploit alignment-free methods to identify proteins that target the same protein via convergent evolution of protein interaction domains. Such convergence might not be evident in primary sequence but could be revealed by more sophisticated methods that probe physiochemical properties of amino acids such as mass, volume, surface area, hydrophilicity, hydrophobicity, isoelectric point, transfer of energy solvent to water, refractivity, non-polar surface area, and frequencies of alpha-helix, beta-sheet, and reverse turn. In this way, we could utilize information from bacterial effectors to accelerate understanding of effectors from organisms that contain more complex effectoromes and are less tractable from an experimental standpoint.

We approached this problem by testing whether a bioinformatics-driven approach could identify effector proteins from *H*. *arabidopsidis* that are similar to the well-studied AvrE1 protein. An alignment-free, PLS method was used because alignment-based methods, such as profile HMMs, become unreliable when sequence similarity drops below 40% [74]. Some families of effector proteins, such as the AvrE-family, are highly divergent and have low sequence identity even though they likely still share similar structures, biochemical properties, and functions. In such cases, obtaining reliable alignments among these protein sequences is extremely difficult, and alignment-based methods such as BLAST, PSI-BLAST (position specific iterative BLAST [75]), and profile HMMs would fail to identify these proteins from databases.

Using PLS alignment-free methods [56], we identified nine candidate effector genes from *H*. *arabidopsidis* predicted to be similar to AvrE-family type III effector proteins. The structures of these nine proteins were predicted using the I-TASSER server and compared with the AvrE1 predicted protein structure using DaliLite. Five of the nine *Hpa* proteins were predicted to share structural similarity with AvrE1. HaRxL23 was selected for further study because of its high Z-score (Fig 1, Table 1) and because it has been experimentally validated as a *bona fide* effector [51]. Moreover, we were intrigued that HaRxL23 is one of the most evolutionarily conserved RXLR effectors in the *Hpa* genome, it is one of only three *Hpa* effector candidates for which homologous genes are recognizable at syntenic loci in *Phytophthora* genomes, and it contributes to virulence in evolutionarily divergent species [39, 51, 66]. Similarly, AvrE-family effectors are among the most conserved bacterial Type III effectors, are broadly distributed among distinct genera of phytopathogenic genera, and contribute to the virulence of these diverse bacteria in divergent host plants [21–27]. Thus, we hypothesize that AvrE1 and HaRxL23 promote virulence by targeting evolutionarily conserved protein(s) in divergent plant species.

As a first step towards testing this hypothesis, we conducted genetic experiments to test for functional similarity between HaRxL23 and AvrE1. Importantly, we demonstrated that HaRxL23 could rescue the reduced bacterial speck lesion phenotype of the *avrE1* mutant, demonstrating that HaRxL23 can genetically complement the avrE1 mutant. We also determined that, in older *Arabidopsis* plants, both effectors were successful in suppressing *Pph* 3121-induced callose deposition when delivered by *Pph*. PTI readouts, such as the callose deposition suppressed by AvrE1 and HopM1, are both SA-dependent [24] and SA-independent [76, 77]. It is possible that HaRxL23 targets similar conserved SA-mediated immunity pathways in the host as a possible virulence mechanism. Accordingly, *Arabidopsis* plants expressing transgenic HaRxL23 display reduced induction of the SA-responsive gene PR-1 during infection by *Hpa* [51].

Another set of experiments was directed towards understanding whether HaRxL23 and AvrE1 could induce cell death when delivered by *Pph* in *Arabidopsis*. It has been previously shown that AvrE1 induces cell death when delivered from a high dose of bacteria infiltrated into leaves of susceptible tobacco (*Nicotiana tabacum*) or tomato plants [66]. We delivered HaRxL23 and AvrE1 from *Pseudomonas phaseolicola* (*Pph*) 3121 and found that each induced cell death in the leaves of young *Arabidopsis* plants. Previous studies had hypothesized a strong correlation between suppression of basal defense and R protein-independent cell-death promotion by bacterial effectors AvrE1 and HopM1 [66], where both these effectors restored basal resistance suppression to the ΔCEL mutant and produced a delayed necrosis when transiently expressed in the host. The authors proposed that the recognition event triggered by AvrE1 could be explained by a mechanism through which the plant perceived strong suppression of PTI by high levels of AvrE1 and responded by triggering cell death. More recently, AvrE1 and HopM1 have been shown to promote water-soaking in the leaf, thereby converting the apoplast into a aqueous environment [16]. The promotion of water-soaking by HopM1 requires the *Arabidopsis* MIN7 protein that regulates vesicle trafficking and is targeted by HopM1. The mechanism through which AvrE1 promotes water soaking is not known. Water-soaking is thought to benefit bacteria by interfering with apoplastic defenses, facilitating nutrient flow, and/or promoting bacterial dissemination outside of the leaf. Considering that the vast majority of oomycete (i.e., hyphae) biomass resides in the apoplast during colonization, oomycetes could benefit similarly from an aqueous environment in the apoplast. Thus, it will be of interest to examine whether the virulence-promoting activities of HaRxL23 are linked to the water status of the apoplast.

As mentioned above, an important, emerging concept in effector biology is that effectors from pathogens as distantly related as bacteria, fungi, oomycetes, and nematodes can perturb the same target proteins/pathways in the host. One example of this is provided by the cysteine-rich protease RCR3 protein from tomato. RCR3 is targeted by effectors from three unrelated phytopathogens: Avr2 from the fungus *Cladosporium fulvum*, EPIC1 & EPIC2B from the oomycete *Phytophthora infestans* and VAP1 from the root nematode *Globodera rostochiensis* [78, 79]. Thus, RCR3 can be thought of as an Achilles’ Heel that fungi, oomycetes and nematodes have evolved independently to target as part of their virulence program.

In a similar vein, but on a much broader scale, Mukhtar et al. [49] undertook a yeast two-hybrid protein interaction study between a fraction of the *Arabidopsis* proteome and effectors from bacteria and *Hpa*. One goal of this screen was to identify effector targets. They identified a set of 18 “core” *Arabidopsis* proteins that were targeted by effectors from bacteria and *Hpa*, and presumably represent pathways that are important for the interaction with both pathogens [49]. A subsequent study extended this approach to identify *Arabidopsis* proteins convergently targeted by effectors from pathogens representing three kingdoms of life: The bacterium *P*. *syringae*, the oomycete *Hpa*, and *Golovinomyces orontii*, a powdery mildew fungus of *Arabidopsis* [48].

Based on these precedents and upon the data in this study, we propose that AvrE1 and HaRxL23 target the same (or similar) protein(s) in *Arabidopsis*. Considering that both effectors are amongst the most highly conserved in their respective pathogen lineages, their target(s) are likely to hold broad importance for plant-bacteria and plant-oomycete interactions. The next step will be directed and unbiased protein interaction assays to identify HaRxL23’s host protein target(s) and thereby directly test the hypothesis that HaRxL23 targets the same *Arabidopsis* proteins as do AvrE-family T3Es. Regulatory subunits of PP2A are one example of a possible shared target between AvrE1 and HaRxL23.

In a broader context, this study has provided an example of how alignment-free approaches can be applied to functional genomic characterization of effector protein, towards the ultimate goals of leveraging insight from model plant pathogens and identifying effectors that have convergently evolved to manipulate high-value host protein targets. The approaches in this paper are not restricted to any particular lineage of pathogen and could be applied to any species for which a set of effector genes has been delineated. Judicious application of these tools provides another approach towards the important and challenging goal of reducing a long list of effector protein candidates to a tractable list of high value leads and to illuminating shared activities and host targets of effectors from divergent pathogens.

## Supporting information

S1 Fig Structural predictions of PsAvh73 and AvrE1. (DOCX)

S1 Table *H*. *arabidopsidis* effector genes used in this study. (DOCX)

S2 Table 12 AvrE protein sequences used for training the methods. (DOCX)

S3 Table 12 Non-AvrE protein sequences used for training the methods. (DOCX)

S4 Table All protein candidates identified from *Hyaloperonospora arabidopsidis* genome by the three methods. (DOCX)

S5 Table Pairwise structural comparison of AvrE1 and the nine protein candidates from *H*. *Arabidopsidis* by DaliLite. (DOCX)

S6 Table Structural templates used by ITASSER to model AvrE and HaRxL23 structures. (DOCX)

S7 Table Table of primers used in this study. (DOCX)
