# Supplementary material for: Application of alignment-free bioinformatics methods to identify an oomycete protein with structural and functional similarity to the bacterial AvrE effector protein
Source: PLoS One. 2018 Apr 11;13(4):e0195559. doi: 10.1371/journal.pone.0195559 (PMC5895030; doi:10.1371/journal.pone.0195559)
Supplement: S1 Fig — (DOCX) [file pone.0195559.s001.docx]

**S1 Fig.** **Structural predictions of PsAvh73 and AvrE1.** (A) Predicted structure of AvrE1 by I-TASSER (B) Predicted structure of PsAvh73 by I-TASSER (C) Superimposed structure of PsAvh73 on AvrE1 by DaliLite. Structure of PsAvh73 is shown in red.
