# Supplementary material for: Application of alignment-free bioinformatics methods to identify an oomycete protein with structural and functional similarity to the bacterial AvrE effector protein
Source: PLoS One. 2018 Apr 11;13(4):e0195559. doi: 10.1371/journal.pone.0195559 (PMC5895030; doi:10.1371/journal.pone.0195559)
Supplement: S1 Table — (DOCX) [file pone.0195559.s002.docx]

**S1 Table. *H. arabidopsidis* effector genes used in this study.** Accession numbers are from FungiDB.org; see also Supplementary Information from ([1](#_ENREF_1)) for a complete table of predicted proteins from 134 high-confidence *Hpa* RxLR gene candidates.

1. Baxter L, Tripathy S, Ishaque N, Boot N, Cabral A, Kemen E, et al. Signatures of adaptation to obligate biotrophy in the *Hyaloperonospora arabidopsidis* genome. Science. 2010; 330:1549-51.

| **No** | **Gene** | **Accession number** |
| --- | --- | --- |
| 1 | HaRxL23 | [HpaG803062:RNA-p1](http://fungidb.org/fungidb/showRecord.do?name=TranscriptRecordClasses.TranscriptRecordClass&project_id=FungiDB&source_id=HpaG803062%3ARNA&gene_source_id=HpaG803062) |
| 2 | HaRxL33 | [HpaG805623:RNA-p1](http://fungidb.org/fungidb/showRecord.do?name=TranscriptRecordClasses.TranscriptRecordClass&project_id=FungiDB&source_id=HpaG805623%3ARNA&gene_source_id=HpaG805623) |
| 3 | HaRxL71 | [HpaG805267:RNA-p1](http://fungidb.org/fungidb/showRecord.do?name=TranscriptRecordClasses.TranscriptRecordClass&project_id=FungiDB&source_id=HpaG805267%3ARNA&gene_source_id=HpaG805267) |
| 4 | HaRxL94 | [HpaG803112:RNA-p1](http://fungidb.org/fungidb/showRecord.do?name=TranscriptRecordClasses.TranscriptRecordClass&project_id=FungiDB&source_id=HpaG803112%3ARNA&gene_source_id=HpaG803112) |
| 5 | HaRxL120 | [HpaG810298:RNA-p1](http://fungidb.org/fungidb/showRecord.do?name=TranscriptRecordClasses.TranscriptRecordClass&project_id=FungiDB&source_id=HpaG810298%3ARNA&gene_source_id=HpaG810298) |
| 6 | HaCRN9 | [HpaG811556:RNA-p1](http://fungidb.org/fungidb/showRecord.do?name=TranscriptRecordClasses.TranscriptRecordClass&project_id=FungiDB&source_id=HpaG811556%3ARNA&gene_source_id=HpaG811556) |
| 7 | HaCRN10 | [HpaG811555:RNA-p1](http://fungidb.org/fungidb/showRecord.do?name=TranscriptRecordClasses.TranscriptRecordClass&project_id=FungiDB&source_id=HpaG811555%3ARNA&gene_source_id=HpaG811555) |
| 8 | HaCRN12 | [HpaG810095:RNA-p1](http://fungidb.org/fungidb/showRecord.do?name=TranscriptRecordClasses.TranscriptRecordClass&project_id=FungiDB&source_id=HpaG810095%3ARNA&gene_source_id=HpaG810095) |
| 9 | HaCRN14 | [HpaG814480:RNA-p1](http://fungidb.org/fungidb/showRecord.do?name=TranscriptRecordClasses.TranscriptRecordClass&project_id=FungiDB&source_id=HpaG814480%3ARNA&gene_source_id=HpaG814480) |
