# Supplementary material for: Application of alignment-free bioinformatics methods to identify an oomycete protein with structural and functional similarity to the bacterial AvrE effector protein
Source: PLoS One. 2018 Apr 11;13(4):e0195559. doi: 10.1371/journal.pone.0195559 (PMC5895030; doi:10.1371/journal.pone.0195559)
Supplement: S2 Table — (DOCX) [file pone.0195559.s003.docx]

**S2 Table. 12 AvrE protein sequences used for training the methods**

| No | Protein | Accession number |
| --- | --- | --- |
| 1 | AvrE1 | WP_011167987 |
| 2 | dspEF | AAC06134 |
| 3 | AvrE | ZP_00125692 |
| 4 | AvrE | AAT96164 |
| 5 | AvrE | ABA47296 |
| 6 | AvrE | AAT96307 |
| 7 | AvrE | WP_011093674 |
| 8 | DspE | AAC04850 |
| 9 | DspE | AAS45452 |
| 10 | DspE | AAF76343 |
| 11 | WtsE | AAG01467 |
| 12 | AvrE | AAK74145 |
