# Supplementary material for: Application of alignment-free bioinformatics methods to identify an oomycete protein with structural and functional similarity to the bacterial AvrE effector protein
Source: PLoS One. 2018 Apr 11;13(4):e0195559. doi: 10.1371/journal.pone.0195559 (PMC5895030; doi:10.1371/journal.pone.0195559)
Supplement: S3 Table — (DOCX) [file pone.0195559.s004.docx]

**S3 Table. 12 Non-AvrE protein sequences used for training the methods**

| No | Protein | Accession number |
| --- | --- | --- |
| 1 | PIP1-4 | NP_567178 |
| 2 | Hyaluronan synthase | P0C0H1 |
| 3 | Lipoxygenase 1 | XP_015632769 |
| 4 | MCM complex subunit Mcm2 | NP_595477 |
| 5 | Ubiquinone biosynthesis | OJI35312 |
| 6 | trimethylguanosine synthase | NP_473430 |
| 7 | NCAP_CVM1 | P18446 |
| 8 | methylmalonyl-CoA | WP_003407587 |
| 9 | glutamate | WP_005902800 |
| 10 | A-kinase anchor protein 11 | NP_057332 |
| 11 | copper-containing nitrite reductase | WP_041987461 |
| 12 | MYC2 | Q39204 |
