# Supplementary material for: Application of alignment-free bioinformatics methods to identify an oomycete protein with structural and functional similarity to the bacterial AvrE effector protein
Source: PLoS One. 2018 Apr 11;13(4):e0195559. doi: 10.1371/journal.pone.0195559 (PMC5895030; doi:10.1371/journal.pone.0195559)
Supplement: S4 Table — (DOCX) [file pone.0195559.s005.docx]

**S4 Table. All protein candidates identified from *Hyaloperonospora arabidopsidis* genome by the three methods.**

| **Accession number** | **PLS-AA** | **PLS-DIP** | **PLS-ACC** |
| --- | --- | --- | --- |
| HaRxL23 | 0.98 | 0.99 | 0.95 |
| HaRxL33 | 0.99 | 1.00 | 1.00 |
| HaRxL71 | 1.00 | 0.98 | 0.94 |
| HaRxL94 | 0.94 | 0.96 | 0.94 |
| HaRxL120 | 0.94 | 0.97 | 1.01 |
| HaCRN9 | 0.95 | 0.99 | 1.00 |
| HaCRN10 | 0.97 | 1.00 | 1.00 |
| HaCRN12 | 0.98 | 0.97 | 0.95 |
| HaCRN14 | 0.94 | 0.96 | 0.98 |
| HaRxL27 | 0.67 | 0.65 | 0.90 |
| HaRxL42 | 0.92 | 0.43 | 0.88 |
| HaRxL62 | 0.89 | 0.96 | 0.77 |
| HaRxL68 | 0.93 | 0.88 | 0.56 |
| HaRxL103 | 0.67 | 0.72 | 0.98 |
| HaRxL117 | 0.94 | 0.94 | 0.43 |
| HaRxL191 | 0.96 | 0.90 | 0.78 |
| HaRxL193 | 0.34 | 0.97 | 0.84 |
| HaCRN15 | 0.88 | 0.45 | 0.96 |
| HaRxL3 | 0.88 | 0.66 | 0.93 |
| HaRxL17 | 1.00 | 0.54 | 0.86 |
| HaRxL22 | 0.77 | 0.91 | 0.93 |
| HaRxL37 | 0.89 | 0.42 | 0.96 |
| HaRxL46 | 0.98 | 0.90 | 0.76 |
| HaRxL77 | 0.93 | 0.97 | 0.45 |
| HaRxL107 | 0.98 | 0.63 | 0.76 |
| CBI63255 | 0.92 | 0.25 | 0.76 |
| AEF57462 | 0.90 | 0.31 | 0.43 |
| AEF57457 | 0.95 | 0.54 | 0.55 |
| AIF71151 | 0.97 | 0.61 | 0.26 |
