# Supplementary material for: Application of alignment-free bioinformatics methods to identify an oomycete protein with structural and functional similarity to the bacterial AvrE effector protein
Source: PLoS One. 2018 Apr 11;13(4):e0195559. doi: 10.1371/journal.pone.0195559 (PMC5895030; doi:10.1371/journal.pone.0195559)
Supplement: S5 Table — (DOCX) [file pone.0195559.s006.docx]

**S5 Table. Pairwise structural comparison of AvrE1 and the nine protein candidates from *H. Arabidopsidis* by DaliLite**

| No | ID | Z scores |
| --- | --- | --- |
| 1 | HaRxL23 | Z = 6.7 |
| 2 | HaRxL33 | Z = 4.4 |
| 3 | HaRxL71 | Z = 4.7 |
| 4 | HaRxL94 | Z = 5.7 |
| 5 | HaRxL120 | No hit |
| 6 | HaCRN9 | No hit |
| 7 | HaCRN10 | Z = 2.2 |
| 8 | HaCRN12 | No hit |
| 9 | HaCRN14 | No hit |
