# Supplementary material for: Application of alignment-free bioinformatics methods to identify an oomycete protein with structural and functional similarity to the bacterial AvrE effector protein
Source: PLoS One. 2018 Apr 11;13(4):e0195559. doi: 10.1371/journal.pone.0195559 (PMC5895030; doi:10.1371/journal.pone.0195559)
Supplement: S6 Table — (DOCX) [file pone.0195559.s007.docx]

| **S6 Table. Structural templates used by ITASSER to model AvrE and HaRxL23 structures** | | |
| --- | --- | --- |
| **AvrE templates** | **HaRxL23 templates** |  |
| 4IGL: Structure of the RHS-repeat containing BC component of the secreted ABC toxin complex from Yersinia entomophaga | 5L7S2: Crystal structure of RXLR effector PexRD54 from Phytophthora infestans |  |
| 5A9Q: Human nuclear pore complex | 2LC2: Solution structure of the RXLR effector P. capsici AVR3a4 |  |
| 5YFP: Cryo-EM Structure of the Exocyst Complex | 2RGH: Structure of Alpha-Glycerophosphate Oxidase from Streptococcus sp.: A Template for the Mitochondrial Alpha-Glycerophosphate Dehydrogenase |  |
