# Supplementary material for: Application of alignment-free bioinformatics methods to identify an oomycete protein with structural and functional similarity to the bacterial AvrE effector protein
Source: PLoS One. 2018 Apr 11;13(4):e0195559. doi: 10.1371/journal.pone.0195559 (PMC5895030; doi:10.1371/journal.pone.0195559)
Supplement: S7 Table — (DOCX) [file pone.0195559.s008.docx]

**S7 Table. Table of primers used in this study.**

| HaRxL23 NOSP | CACCATGGCAACGTCTACCGATCTGA |
| --- | --- |
| HaRxL23 NS | GGCGTCGACGTGCTTTAGGC |
| HaRxL23 S | CTAGGCGTCGACGTGCTTTA |
| Avh73 NOSP | GCTTCTGCTTCTTCAGAGCTCGTCGC |
| Avh73 NS | AGGCGGCTTTGCCTTCGAGG |
| Avh73 S | GTATTTGCCGTACTGGGTGA |
| pEDV6 Fwd | GGCACCCCAGGCTTTACACTTTATG |
| M13 Fwd | GTAAAACGACGGCCAGTG |
| M13 Rev | GGAAACAGCTATGACCATG |
